# The efficacy and safety of electrical acupoint stimulation (EAS) for knee osteoarthritis (KOA): A GRADE-assessed systematic review, meta-analysis and trial sequential analysis

**DOI:** 10.1371/journal.pone.0331568

**Published:** 2025-09-25

**Authors:** Guangyi Yang, Rong Zhang, Botan Xu, Yi Yang, Yuxuan Wang, Wenjing Xu, Pengwei Li, Aotian Yu, Simin Ning, Qixuan Fu, Jianfeng Tu

**Affiliations:** 1 School of Acupuncture-Moxibustion and Tuina, Beijing University of Chinese Medicine, Beijing, China; 2 Graduate School, Beijing University of Chinese Medicine, Beijing, China; 3 The Third Clinical Medical College, Beijing University of Chinese Medicine, Beijing, China; 4 School of Nursing, Beijing University of Chinese Medicine, Beijing, China; 5 School of Chinese Medicine, Beijing University of Chinese Medicine, Beijing, China; The Affiliated Changzhou No 2 People's Hospital of Nanjing Medical University, CHINA

## Abstract

Electrical acupoint stimulation (EAS) is proposed as a potentially beneficial treatment for patients suffering from knee osteoarthritis (KOA). This systematic review and meta-analysis aims to assess the assess the effectiveness and safety of EAS for KOA. To identify eligible RCTs, a systematic search for eligible RCTs is conducted through 6 November 2024 in 12 electronic literature databases, utilizing relevant keywords. The analysis applies a random-effects model to compute RRs and 95% CIs for the dichotomous outcome, alongside the SMD and 95% CIs for continuous outcomes. This meta-analysis includes 63 RCTs with a total of 6475 participants. The pooled analysis reveals that EAS increases the overall response rate by 19.5% (RR: 1.195, 95% CI: 1.130 to 1.264, P < 0.001; low certainty). For continuous outcomes, EAS produces large effects on WOMAC total score (SMD = −1.72, 95% CI −2.19 to −1.24; P < 0.001; low certainty), VAS score (SMD = −1.92, 95% CI −2.44 to −1.39; P < 0.001; very low certainty), WOMAC stiffness (SMD = −1.12, 95% CI −1.65 to −0.59; P < 0.001; low certainty) and Lysholm score (SMD = 1.10, 95% CI 0.47 to 1.73; P = 0.001; low certainty). It yields medium effects on WOMAC pain (SMD = −0.74, 95% CI −1.10 to −0.37; P < 0.001; low certainty), Lequesne index (SMD = −0.70, 95% CI −0.97 to −0.42; P < 0.001; low certainty) and WOMAC function (SMD = −0.51, 95% CI −0.97 to −0.05; P = 0.03; low certainty). There are no significant effects on peak quadriceps torque (SMD = 0.21, 95% CI −0.42 to 0.83; P = 0.518; low certainty) or knee range of motion (SMD = 0.66, 95% CI −0.07 to 1.38; P = 0.077; low certainty). Trial sequential analysis indicates that the required information size is met. This review suggests that EAS may be an effective and relatively safe adjunctive option for KOA; however, the certainty of the evidence is low due to substantial heterogeneity and potential biases. Higher-quality, rigorously designed RCTs with standardized reporting are needed to confirm these findings.

## 1. Introduction

Knee osteoarthritis (KOA) is one of the most common musculoskeletal disorders, contributing significantly to the global disability burden, particularly among the elderly [1]. According to the World Health Organization (WHO), approximately 595 million people worldwide suffer from osteoarthritis, with KOA affecting approximately 365 million people [2,3]. In China, approximately 37.5% to 46.6% of individuals over the age of 60 are affected by osteoarthritis, and the prevalence increases to 80% in those over 75 years old [4–7]. The condition is characterized by degenerative joint changes that cause symptoms such as persistent pain, stiffness, reduced flexibility, and a significant decline in quality of life. These symptoms not only affect physical health but also lead to substantial psychological and socio-economic impacts [2,8]. Despite the availability of treatments like medication and physical therapy, these often provide limited long-term relief, and their side effects complicate patient care [9–11]. Surgical interventions, though effective for some, come with their own set of risks and limitations such as the potential post-operative complications and high costs [12]. Therefore, there is a pressing need to explore safer and side-effect-free therapeutic alternatives.

Electrical acupoint stimulation (EAS), a novel approach that combines traditional acupuncture principles with modern electrotherapy, presents a promising avenue for KOA treatment [13]. As the two core forms of EAS, electroacupuncture (EA) involves inserting needles into acupoints followed by electrical stimulation, whereas transcutaneous electrical acupoint stimulation (TEAS) delivers similar stimulation non-invasively via surface electrodes. EA is hypothesized to relieve pain and enhance function by modulating peripheral nerve excitability, activating endogenous opioid pathways, and improving local blood circulation while TEAS primarily acts through regulating neuroendocrine activity, reducing oxidative stress, and modulating immune and vascular functions [14–16]. Recent studies have explored the use of EAS for various musculoskeletal conditions and have highlighted its potential benefits in pain management and functional recovery [17,18]. Meanwhile, compared to pharmacological treatments, EAS is considered to have fewer side effects and potentially more sustained therapeutic benefits [19]. However, the clinical application of EAS is limited by the uncertainty in current research outcomes arising from the heterogeneity of study designs, variability in treatment protocols, and inconsistency in outcome measures [20]. Moreover, although EA and TEAS have been evaluated separately in prior studies, few systematic reviews have synthesized the evidence for EAS as a unified category. This fragmented approach limits our understanding of EAS’s overall clinical utility. Furthermore, previous meta-analyses rarely incorporated rigorous methods such as GRADE and trial sequential analysis (TSA) to assess the certainty and conclusiveness of findings, underscoring the need for a more comprehensive and methodologically robust evaluation. Therefore, as a relatively new approach, the clinical application of EAS for KOA requires further rigorous research to validate its effectiveness, address treatment variability, and determine its role in the management of KOA.

This study aims to perform a systematic review and meta-analysis to evaluate the efficacy and safety of EAS in treating KOA. Evidence from RCTs is systematically synthesized to determine factors that may affect the clinical outcomes of EAS. Furthermore, this study will examine the evidence quality using GRADE and apply TSA to assess the robustness of the findings. By addressing methodological limitations in prior reviews and integrating fragmented evidence, this study provides more reliable guidance for clinical decision-making regarding the role of EAS in KOA management.

## 2. Methods

### 2.1. Protocol and registration

This investigation is conducted following the guidelines set forth by the Preferred Reporting Items for Systematic Reviews and Meta-Analyses (PRISMA, S1 Table), and the protocol for this review has been registered with PROSPERO (CRD42024537766).

### 2.2. Inclusion and exclusion criteria

Eligible publications are selected based on the PICOS criteria.

#### 2.2.1. Participant.

Inclusion criteria do not restrict age, gender, or race; however, we exclude specific populations such as pregnant or lactating women, individuals with implanted devices, those suffering from allergies, and critically ill patients. Eligibility requires that studies focus on KOA and utilize officially recognized or validated diagnostic criteria. Consequently, studies involving other forms of arthritis (e.g., rheumatoid, gouty, acute septic, metabolic) are excluded.

#### 2.2.2. Intervention.

This review focuses on RCTs investigating the effectiveness of EAS, comprising EA and TEAS, as the primary treatment for KOA. The minimum acceptable parameters for inclusion are based on ISO 24571:2022 standards and include: (i) pulse frequency ≥2 Hz; (ii) pulse duration <10 ms; (iii) output current ≤1 mA at 500 Ω; (iv) output voltage ≤100 V peak; and (v) use of at least 4 acupoints. Exclusion criteria encompass studies that: (i) administer conventional treatments alongside EAS to participants; (ii) apply EAS in both the intervention and control groups; (iii) fail to provide a comprehensive EAS methodology, detailing parameters such as frequency, duration, and acupuncture points.

#### 2.2.3. Control.

Baseline characteristics such as age, sex, and the Kellgren & Lawrence (KL) classification stage show no significant differences between the intervention and control groups. Furthermore, studies where additional treatments (e.g., medication, physical therapy) are administered, provided these are consistent across all participant groups in dosage.

#### 2.2.4. Outcome.

The primary outcome, evaluated at the end of the intervention, is the overall response rate (dichotomous). Secondary outcomes include key osteoarthritis indices assessed using the Western Ontario and McMaster Universities Osteoarthritis Index (WOMAC) (continuous), Lequesne Algofunctional Index (continuous), Visual Analogue Scale (VAS) (continuous), and Lysholm Knee Scoring Scale (continuous). Additionally, objective measures including peak muscle torque (continuous) and range of motion (ROM) (continuous) are considered.

#### 2.2.5. Study design.

Only RCTs, regardless of their design (parallel or crossover), are included. Exclusion criteria include: (i) non-clinical research such as animal studies, meta-analyses, or reviews; (ii) non-RCT studies like retrospective studies, cohort studies, case reports, or observational studies; (iii) pilot studies with total participants fewer than 40.

### 2.3. Data sources and search strategy

Under the supervision of senior methodologist W.X., author R.Z. designed and executed the search strategy for multiple databases and conduct a comprehensive search across 12 databases, without language restrictions, from inception through 6 November 2024. These databases include the Cochrane Central Register of Controlled Trials (CENTRAL), PubMed, Excerpta Medica Database (EMBASE), Web of Science (WOS), Google Scholar, OpenSIGLE, ProQuest Dissertation & Theses Database, Scopus, Chinese Scientific Journal Database (VIP), China Biomedical Literature Service System (SinoMed), WanFang Database, and China National Knowledge Infrastructure (CNKI). The search term is comprised of two components: population and intervention. The search strategy for PubMed, used as a template for English-language database searches, is provided in Table 1. The strategy for SinoMed, serving as a template for Chinese-language database searches, is presented in S2 Table. Similar but adaptive search strategies are applied to other electronic databases. Furthermore, a manual search is performed on the reference lists of chosen articles and additional published systematic reviews.

**Table 1:** The search strategy for PubMed database.

Strategy	Number	Search terms
Population	#1	(Knee* [MeSH Terms] OR Knee* Joint [MeSH Terms])
	#2	(Osteoarthritis [MeSH Terms] OR Arthrit* OR Degenerative Arthrit* OR Arthrit*, Degenerative)
	#3	#1 AND #2
	#4	(Osteoarthritis, Knee* [MeSH Terms] OR Knee* Osteoarthritis OR KOA OR Osteoarthritis of Knee* OR Degenerative Joint Disease Knee* OR OA, Knee* OR Ostarthritis, Knee* OR Degenerative Arthritis, Knee* OR Degenerative Arthritis of Knee*)
	#5	#3 OR #4
Intervention	#6	(Electric* Stimulation* [MeSH Terms] OR Electric Stimulation Therap* [MeSH Terms] OR Stimulation, Electric* OR Therap*, Electric Stimulation OR Electrotherap*)
	#7	(Acupuncture Point* [MeSH Terms] OR Point*, Acupuncture OR Acupoint*)
	#8	#6 AND #7
	#9	(Electroacupuncture OR Transcutaneous Electrical Acupoint Stimulation)
	#10	#8 OR #9
	**#5 AND #10**	

### 2.4. Study selection and data extraction

Two reviewers (R.Z. and Y.W.) independently screened the titles and abstracts of articles following a pilot calibration exercise. Duplicate records are removed through EndNote 20’s (Available at: https://endnote.com/) automated deduplication (title/year/journal match) with manual verification of 10% randomly selected entries. Full-text screening employed a standardized decision tree, categorizing exclusions as: population mismatch, intervention mismatch, comparator non-conformity, outcome irrelevance, or study design exclusion. The selection process is documented via PRISMA 2020 flow diagram (http://www.prismastatement.org/), detailing identification, screening, and inclusion stages. Data extraction is conducted by two independent reviewers (G.Y. and Y.Y.) using a pre-validated Excel form structured per Cochrane Handbook Chapter 7.6 (https://training.cochrane.org/handbook), capturing: (i) Study identifiers (title, author, DOI, funding source); (ii) Study specifics (trial design, randomization method, sample size, duration of treatment, morbidity period); (iii) Population (age, sex, diagnostic criteria, group sizes); (iv) Intervention (EAS parameters, comparator details); (v) Outcomes (measurement instruments, timepoints, blinded assessment status, adverse events). Any disagreements during this process are resolved through discussion and, if unresolved, by consulting a third reviewer (W.X.) for final arbitration. In instances where patient-reported outcome is not detailed in the manuscript, the original authors are contacted via email for further information.

### 2.5. Addressing missing data

When encountering trials with missing or insufficient data, we contact the respective authors to obtain more comprehensive information. If this approach proves unfeasible, we analyze the available data and conduct a sensitivity analysis to help us assess the potential influence of any information gaps on the overall results of our meta-analysis. In cases where studies report medians and ranges, the means and standard deviations are estimated using established formulas proposed by Hozo et al. (2005) [21] and Wan et al. (2014) [22], as follows:


$$\begin{array}{c} \overset{―}{x}=\frac{a+2m+b}{\text{4}} \end{array}$$ (1)

$$\begin{array}{c} S^{2}=\frac{1}{\text{1}2}\left\{\frac{(a+2m+b{)}^{2}}{\text{4}}+(b-a{)}^{2}\right\} \end{array}$$ (2)

where a and b represent the minimum and maximum of the range, respectively, and m is the median.

### 2.6. Methodological quality assessment

Two reviewers (B.X. and R.Z.) independently evaluate the methodological quality of included studies using the Cochrane Risk of Bias Tool 2.0 (RoB 2.0) [23]. The tool evaluates seven critical domains grading them as low, high, or unclear risk: (i) randomization sequence generation, (ii) allocation concealment, (iii) blinding of participants and personnel, (iv) blinding of outcome assessment, (v) incomplete outcome data, (vi) selective reporting, and (vii) other bias. Subsequently, trials are classified as low, high, or moderate quality based on the criteria proposed by Zhao [24], which prioritize the domains of randomization and allocation concealment: (i) low quality for high bias risk in randomization or allocation concealment; (ii) high quality for low bias risk in both and low/unclear risk in all other aspects; (iii) moderate quality if criteria for neither high nor low quality are met. This classification is adopted for its explicit emphasis on methodological safeguards that are recognized in the Cochrane Handbook as fundamental to preserving internal validity and reducing the risk of bias. Risk-of-bias judgments are visualized using Review Manager (version 5.3), presenting domain-specific assessments in traffic light plots and summary graphs. Discrepancies between reviewers are resolved through iterative discussion, with unresolved cases adjudicated by a senior methodologist (W.X.).

### 2.7. Assessment of publication bias

For outcomes involving ≥10 studies, contour-enhanced funnel plots with statistical significance contours are generated to visually assess asymmetry [25,26]. Subsequently, Egger’s or Harbord’s regression tests (p < 0.10 threshold) are applied to quantify small-study effects, supplemented by trim-and-fill analysis to estimate potential missing studies.

### 2.8. Quality of evidence according to outcome measures

Evidence quality is graded using the Grading of Recommendations, Assessment, Development, and Evaluation (GRADE) framework across five domains: risk of bias, inconsistency, indirectness, imprecision, and publication bias [27]. Outcomes are classified as high, moderate, low, or very low certainty.

### 2.9. Statistical analysis

#### 2.9.1. Data synthesis and analysis.

Between-study heterogeneity is quantified using I² and Cochran’s Q test. A value of I^2^ > 50% and P < 0.1 for the Q test is considered as significant heterogeneity between studies [28]. Random-effects models (DerSimonian-Laird method) are preferentially applied when I^2^ ≥ 50% or P ≤ 0.1, with fixed-effects models reserved for homogeneous subgroups (I² < 50% and P > 0.1). Continuous outcomes are synthesized as Cohen standardized mean differences (SMDs) with 95% confidence intervals (CIs), while dichotomous outcomes are pooled and detailed as Mantel-Haenszel risk ratios (RRs). SMDs are interpreted according to Cohen’s thresholds: values of |0.2| indicate a small effect, |0.5| a medium effect, and |0.8| or greater a large effect [29]. To investigate potential sources of heterogeneity, we conduct a tiered analytical approach as prespecified in the PROSPERO protocol (CRD42024537766). Initially, leave-one-out sensitivity analysis is systematically performed to identify influential outlier studies. Then, for primary outcome, subgroup analyses examine 8 clinically relevant variables: (i) number of participants (≥200 and <200), (ii) type of intervention (EA and TEAS), (iii) waveform (dilatational wave and continuous wave), (iv) acupoint count (≥6 and <6), (v) intervention duration (≥4weeks and <4weeks), (vi) needle retention time (≥30minutes and <30minutes), (vii) control group intervention (placebo, physical therapy and medicine), and (viii) follow-up period (≥6months and <6 months). Secondary outcomes undergo dual analytical strategies: (a) subgroup analyses focused on intervention modality and treatment duration to assess consistency across outcome types; (b) multivariable meta-regression incorporating 4 continuous predictors: needle retention time (minutes), frequency of intervention (times/week), total treatment weeks, and follow-up duration (months). Statistical synthesis is conducted using STATA software (v17.0, Stata/M.P., College Station, Texas, USA).

#### 2.9.2. Additional analysis.

To assess the adequacy and power of the cumulative data for outcome evaluation, we conduct trial sequential analysis using TSA software (version 0.9.5.10). This analysis aims to maintain a 5% risk of type I error and ensure 80% statistical power [30].

## 3. Results

### 3.1. Study selection

**Fig 1:**
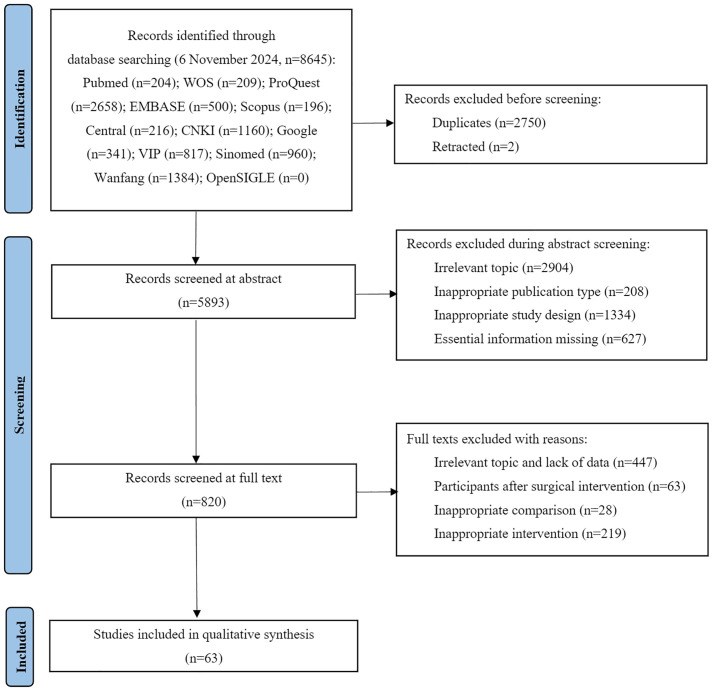
Summary of studies identification and selection according to the PRISMA flow diagram.

Fig 1 illustrates the procedure for identifying and screening publications for inclusion in the meta-analysis. Initially, a total of 8645 publications are identified using the predefined search strategy. After removing duplicates, the number is reduced to 5894 relevant articles. Subsequent screening of titles and abstracts led to the selection of 820 articles for full-text review. Following a comprehensive evaluation, 63 original trials [31–93] encompassing a total of 6475 participants, are found to meet the inclusion criteria and are included in the final meta-analysis.

### 3.2. Characteristics of included studies

**Fig 2:**
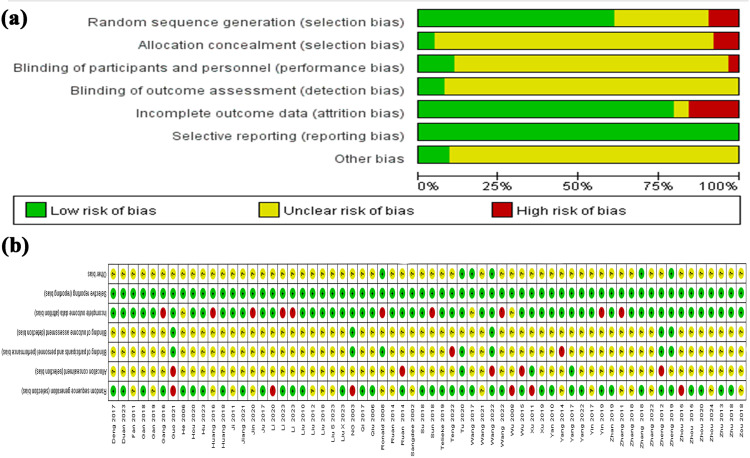
Risk of bias graph and summary.

The characteristics of the 63 publications [31–93] included in this meta-analysis are listed in Table 2, while S3 Table presents the additional study and sample details. These studies are published between 2002 and 2024, and all from Asian, except for one study from Australia [43] and another one from Europe [35]. As for the diagnostic criteria for KOA, the criteria proposed by the Chinese medical association is the most widely used among the 9 criteria with a total of 26 studies [38,40,41,51–53,56,58,59,63,65,67,68,70,73,76–79,83,87–91,93]. All studies are performed on both genders except for 2 studies exclusively performed on male subjects [75,83] and 2 don’t report [53,58]. Sample size ranges from 33 to 722, and the participants’ age ranges from 34 to 76 years. The follow-up period ranges from 0 to 24 weeks, the intervention frequency varied from 2 to 7 times per week, and duration of trials differed from 10 days to 8 weeks across included RCTs. All studies employ a parallel design, with 34 [32,38,44,48,50–54,56,58,59,64,66,69,71–73,75–81,84,85,87–90,92,93] receiving financial support. Additionally, there are 32 studies [31,32,34,37,39,40,44,46,48,50–59,61,64,69,70,72,74,75,79–81,84,90,92] using EAS in monotherapy as the comparator group. Regarding primary interventions, 5 studies [47,60,73,77,82] administer TEAS while the remainder employs EA and 11 studies [31,35,43,47,56,69,70,74,80,81,84] compare it solely with a placebo. Fig 2 presents the risk of bias assessment for the included studies. According to the Cochrane Collaboration’s tools,2 studies [57,80] are classified as “good” quality, while 9 studies [32,40,43,45,48,50,76,78,83] are deemed of “weak” quality.

**Table 2:** Characteristics of included studies in meta-analysis.

Study (Dx)	Study design; Follow-up period (wk)	Sample size		Intervention				Outcome
		IG	CG	Primary intervention	Combined intervention	Intervention frequency	CG	
Tu et al 2021 [80] (b)	RCT; 18	151	146	EA	NR	3/wk	Placebo	1,2,3,4
Zhang et al 2018 [65] (a)	RCT; NR	30	30	EA	Medicine (ShiWei RuXiang capsule)	5/wk	Medicine (Shiwei ruxiang capsule)	1,6
Yang et al 2017 [57] (b)	RCT; NR	30	30	EA	NR	3/wk	Physical therapy	2,3,4,5,6,9
Xu et al 2011 [40] (a)	RCT; NR	16	17	EA	NR	3/wk	Medicine (Glucosamine sulfate)	2,3,4,5
Zhou et al 2015 [50] (b)	RCT; NR	40	40	EA	NR	7/wk	Medicine (Diclofenac sodium)	6,8
Zheng et al 2012 [43] (c)	RCT; 12	17	18	EA	Physical therapy	2/wk	Placebo	6
Wang et al 2022 [83] (a&d)	RCT; NR	34	34	EA	Physical therapy	3/wk	Physical therapy	5,10
Hou et al 2020 [74] (b)	RCT; NR	18	18	EA	NR	5/wk (1/3); 3/wk (2/3)	Placebo	3
Li et al 2020 [76] (a)	RCT; NR	40	40	EA	Medicine (JinGuLian capsule)	6/wk	Medicine (JinGuLian capsule)	1,5,6
Wang et al 2021 [81] (NR)	RCT; 18	30	30	EA	NR	3/wk	Placebo	2,3,4,5
Sangdee et al 2002 [31] (b)	RCT; 8	48	47	EA	NR	3/wk	Placebo	1,2,3,4,5,6,7
Wu et al 2015 [48] (b)	RCT; NR	48	47	EA	NR	3/wk	Medicine (Votalin)	6,9
Liu et al 2018 [63] (a)	RCT; NR	50	50	EA	Medicine (Celecoxib)	3/wk	Medicine (Celecoxib)	5,6
Xu et al 2019 [71] (b)	RCT; NR	100	100	EA	Medicine (Chinese medicine decoction)	5/wk	Medicine (Meloxicam)	2,3,4,5
Gan et al 2018 [59] (a)	RCT; NR	30	30	EA	NR	3/wk	Medicine (Celecoxib)	1,5,6
Gang et al 2016 [51](a)	RCT; NR	43	45	EA	NR	3/wk	Medicine (Meloxicam)	2,3,4,5
Yin et al 2017 [58] (a)	RCT; NR	60	60	EA	NR	3/wk	Medicine (Aminoglucose)	2,3,4,5,
Yin et al 2019 [72] (NR)	RCT; NR	34	34	EA	NR	3/wk	Medicine (Glucosamine sulfate)	1,5,6,9
Ji et al 2011 [39] (b)	RCT; NR	35	35	EA	NR	3/wk	Medicine (Meloxicam)	1,6,7
Ju et al 2017 [54] (b)	RCT; NR	30	30	EA	NR	3/wk	Medicine (Celecoxib)	2,3,4,5,6
He et al 2006 [33] (e)	RCT; NR	12	12	EA	Physical therapy	7/wk	Physical therapy	6
Zhu et al 2013 [44] (b)	RCT; 8	28	27	EA	NR	3/wk	Medicine (Diclofenac sodium)	1,2,3,4,5
Zhang et al 2011 [41] (a)	RCT; NR	32	32	EA	NR	7/wk	Medicine (Celecoxib)	6
Jiang et al 2021 [79] (a&d)	RCT; NR	50	50	EA	NR	7/wk	Medicine (Diclofenac Sodium)	1,5,6
Yang et al 2022 [85] (f)	RCT; NR	40	40	EA	Physical therapy	7/wk	Medicine (Meloxicam)	8,9
Su et al 2018 [62] (b)	RCT; NR	30	30	EA	Medicine (Celecoxib)	7/wk	Medicine (Celecoxib &Glucosamine sulfate)	6,7
Wang et al 2017 [56] (a&b)	RCT; NR	18	18	EA	NR	5/wk (1/3); 3/wk (2/3)	Placebo	2,3,4,5
Te Lie Ke et al 2019 [70] (a&d)	RCT; NR	28	27	EA	NR	NR	Placebo	1,2,3,4,5,6,10
Liu et al 2012 [42] (b)	RCT; NR	32	31	EA	Physical therapy	7/wk	Physical therapy	6,8
Zhang et al 2018 [65] (b)	RCT; NR	39	39	EA	NR	3/wk	Medicine (Meloxicam)	5,6
Huang et al 2018 [61] (NR)	RCT; NR	100	100	EA	NR	7/wk	Physical therapy	1
Deng et al 2017 [53] (a)	RCT; NR	40	40	EA	NR	3/wk	Medicine (Celecoxib)	6
Zhu et al 2018 [66] (NR)	RCT; 8	40	40	EA	Physical therapy	3/wk	Physical therapy	1,6
Gan et al 2019 [68] (a)	RCT; NR	55	55	EA	Medicine (XianLingGuBao capsule)	3/wk	Medicine (Diclofenac Sodium)	1,2,3,4,5,6
Zhou et al 2020 [77] (a)	RCT; NR	33	33	TEAS	Physical therapy	3/wk	Medicine (Diclofenac sodium)	5,6
Yang et al 2014 [47] (b&d)	RCT; NR	30	30	TEAS	Physical therapy	3/wk	Placebo	2,3,4,5
Zhan et al 2019 [73] (a)	RCT; 4	25	25	TEAS	Physical therapy	3/wk	Physical therapy	1,6,8
Teng et al 2022 [82] (NR)	RCT; NR	31	31	TEAS	Physical therapy	3/wk	Medicine (Diclofenac sodium)	5,6
NG et al 2003 [32] (NR)	RCT; NR	40	40	EA	NR	3/wk	Physical therapy	10
Huang et al 2016 [52] (a&d)	RCT; NR	30	30	EA	NR	3/wk	Medicine (Celecoxib)	8
Wang et al 2022 [84] (b)	RCT; 18	30	30	EA	NR	3/wk	Placebo	1,7,10
Fan et al 2011 [38] (a)	RCT; NR	75	73	EA	Physical therapy	3/wk	Medicine (Nimesulide)	1
Ruan et al 2014 [46] (b)	RCT; NR	45	43	EA	Medicine (Glucosamine hydrochloride)	3/wk	Medicine (Glucosamine hydrochloride)	1
Qiu et al 2006 [34] (b)	RCT; NR	30	30	EA	NR	2/wk	Medicine (Diclofenac Sodium)	2,3,4,5
Qi et al 2017 [55] (g)	RCT; NR	65	60	EA	NR	7/wk	Medicine (Glucosamine sulfate)	1
Ruan et al 2014 [45] (b)	RCT; NR	38	34	EA	NR	7/wk	Medicine (Glucosamine hydrochlorid)	1,7
Jin et al 2020 [75] (NR)	RCT;8	45	45	EA	NR	5/wk	Physical therapy	5
Liu et al 2010 [36] (b)	RCT; NR	60	60	EA	Medicine (Sodium hyaluronate)	5/wk	Medicine (Sodium hyaluronate)	1
Guo et al 2021 [78] (a)	RCT;12	361	361	EA	Physical therapy	3/wk	Physical therapy	7
Lv et al 2019 [69] (b)	RCT; NR	145	75	EA	NR	NR	Placebo	5,6
Zou et al 2018 [67] (a)	RCT; NR	50	50	EA	Medicine (7/wkiclofanac Sodium)	7/wk	Medicine (Diclofanac Sodium)	1,2,3,4
Yan et al 2010 [37] (b)	RCT; NR	60	60	EA	NR	7/wk	Physical therapy	8
Ronald et al 2008 [35] (NR)	RCT;4	34	34	EA	Physical therapy	2/wk	Placebo	2,3,4,6
Zhang et al 2022 [86] (h)	RCT; NR	50	50	EA	Physical therapy&Medicine (Sodium hyaluronate)	7/wk	Physical therapy&Medicine (Sodium hyaluronate)	1,5
Sun et al 2018 [60] (b)	RCT; 24	98	96	TEAS	Physical therapy&Medicine (Glucosamine sulfate)	3/wk	Physical therapy&Medicine (Glucosamine sulfate)	1
Zhou et al 2015 [50] (NR)	RCT; NR	30	30	EA	Physical therapy	7/wk	Physical therapy	1
Li et al 2023 [89] (a)	RCT;4	43	45	EA	Physical therapy	5/wk	Medicine (Celecoxib &Glucosamine hydrochloride)	5,6
Liu et al 2023 [91] (a)	RCT; NR	90	90	EA	Medicine (Glucosamine sulfate & Chinese medicine decoction)	7/wk	Medicine (Glucosamine sulfate)	2,3,4,5,8
Duanmu et al 2023 [87] (a)	RCT; NR	40	40	EA	Physical therapy	7/wk	Physical therapy	5,6,9,10
Hu et al 2023 [88] (a)	RCT; NR	30	30	EA	Physical therapy	7/wk	Physical therapy	1,5,6,8,10
Li et al 2023 [90] (a)	RCT;4	30	30	EA	NR	3/wk	Medicine (Celecoxib)	5
Zhou et al 2024 [93] (a)	RCT; NR	81	81	EA	Physical therapy	6/wk	Physical therapy	1,8
Liu et al 2023 [92] (b)	RCT; NR	135	135	EA	NR	5/wk	Physical therapy	1,5,6,7,8,10

Abbreviations: Dx, diagnosis; IG, intervention group; CG, control group; RCT, randomized controlled trial; EA, electroacupuncture; TEAS, transcutaneous electrical acupoint stimulation; NR, not reported; F, Female; d, day; wk, week; mo, month; Dx: a = The Chinese Medical Association (CMA); b = The American College of Rheumatology (ACR); c = International Association for the Study of Pain (IASP); d = The Guidelines for Traditional Chinese Medicine 7/wkiagnosis and Treatment of Knee Osteoarthritis (G-TCM); e = Clinical disease diagnosis based on cure and improvement standards (C7/wk7/wkCIS); f = 7/wkiagnosis and management of knee osteoarthritis: Chinese medicine expert consensus (2015); g = Guidelines for Clinical Research of New Traditional Chinese Medicine; h = Guidelines for the 7/wkiagnosis and Treatment of Knee Osteoarthritis with the Integrated Traditional Chinese and Western Medicine Outcome: 1 = Overall Response Rate; 2 = WOMAC Function Score; 3 = WOMAC Pain Score; 4 = WOMAC Stiffness Score; 5 = WOMAC Total Score; 6 = Visual Analogue Scale (VAS); 7 = Lequesne score; 8 = Lysholm Score; 9 = Peak torque of muscles; 10 = Range of motion of knees.

### 3.3. Primary outcome: Overall response rate

**Fig 3:**
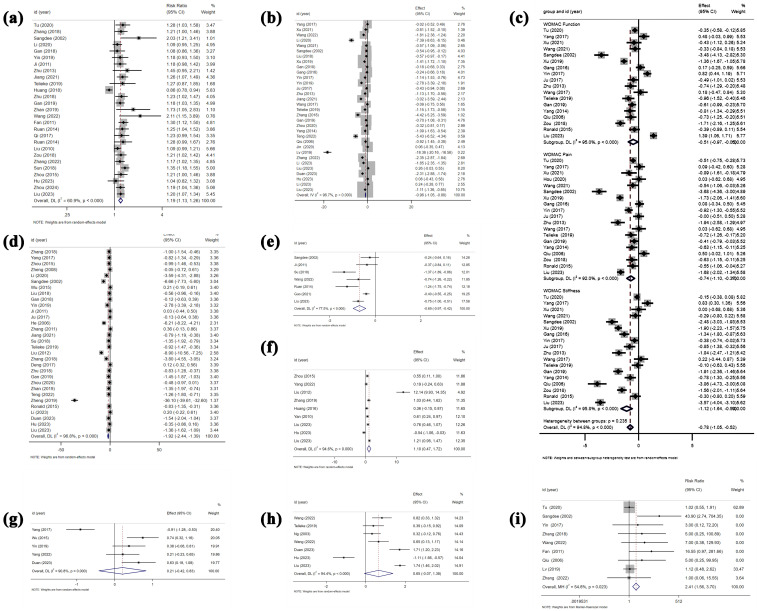
Forest plot detailing effect size and 95%CIs for the outcomes. Positive values indicate a favorable effect of EAS compared for response rate, functional recovery, and muscle strength; negative values indicate greater reductions in symptom scores (e.g., WOMAC pain, WOMAC stiffness, and Visual Analogue Scale). a) Overall response rate (RR); b) WOMAC total score (SMD); c) WOMAC subscales: function, pain, and stiffness (SMD); d) Visual Analogue Scale (VAS, SMD); e) Lequesne index (SMD); f) Lysholm score (SMD); g) Peak muscle torque (SMD); h) Knee range of motion (SMD); i) Incidence of skin and subcutaneous disorders (RR).

**Fig 4:**
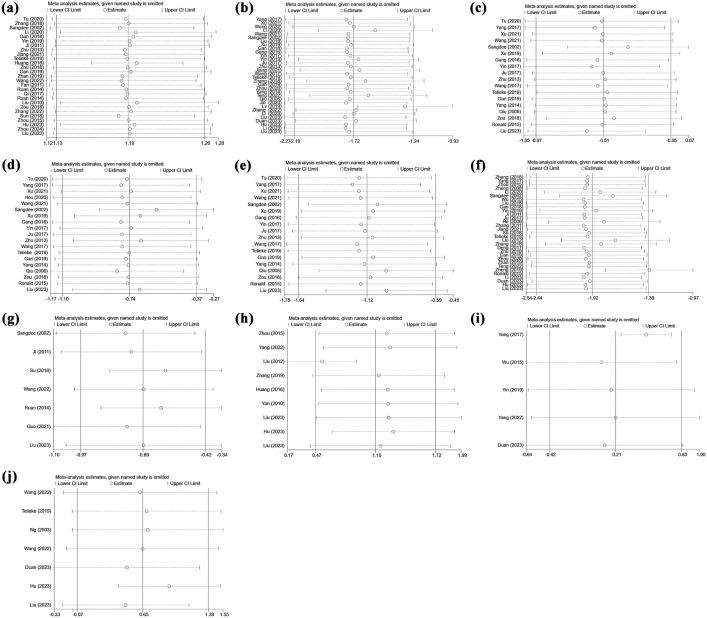
Sensitivity analysis of the synthetic results. a) overall response rate; b) WOMAC Total score; c) WOMAC Function score; d) WOMAC Pain score; e) WOMAC Stiffness score; f) Visual Analogue Scale; g) Lequesne score; h) Lysholm score; i) peak torque of muscle; and j) range motion of knee.

27 RCTs [31,36,38,39,44–46,50,55,59,61,65–68,70,72,73,76,79,84,86,88,92,93], comprising 2950 subjects in total, are included in the analysis to assess the overall response rate. The combination of effect sizes from these 27 studies reveals that EAS increases the overall response rate by 19.5% compared to control treatments (RR: 1.195, 95% CI: 1.130 to 1.264, P < 0.001) (Fig 3a). Moreover, there is evidence of high between-study heterogeneity (I^2^ = 60.9%, P < 0.001). Subgroup analyses, detailed in Table 3, are conducted to identify potential sources of heterogeneity. It is found that heterogeneity exists within subgroups categorized by type of intervention (P = 0.033) and follow-up period (P = 0.038). Sensitivity analysis (Fig 4a) shows that removing any single study results in pooled RRs ranging from 1.189 to 1.208, indicating the robustness of the overall response rate estimate. Visual inspection of the contour-enhanced funnel plot revealed asymmetry (Fig 6a), suggesting potential publication bias. This is further supported by Harbord’s modified test (P = 0.002).

**Table 3:** Subgroup analysis of the effect of EAS on overall response rate.

Subgroup title	No of trials	No of participants	P within group	Risk ratio (95% CI)	Heterogeneity		
					I^2^ (%)	P heterogeneity	P between sub-groups
Overall	27	2518	<0.001	1.198 (1.125, 1.276)	63.6	<0.001	–
No of participants							
≥200	5	1015	0.132	1.157 (0.957, 1.397)	89.5	<0.001	0.765
<200	22	1935	<0.001	1.191 (1.145, 1.239)	0.8	0.449	
Type of intervention							
EA	25	2706	<0.001	1.180 (1.116, 1.249)	58.8	<0.001	0.033^*^
TEAS	2	244	<0.001	1.377 (1.209, 1.568)	0	0.352	
Waveform							
CW	7	788	<0.001	1.200 (1.124, 1.282)	0	0.971	0.407
7/wkW	13	1464	0.002	1.146 (1.051, 1.251)	70.6	<0.001	
Acupoint count							
≥6	22	2290	<0.001	1.168 (1.102, 1.239)	59.1	<0.001	0.107
<6	4	466	0.004	1.444 (1.123, 1.855)	50.0	0.112	
Intervention duration							
≥4wk	21	2245	<0.001	1.187 (1.101, 1.278)	66.9	<0.001	0.356
<4wk	6	705	<0.001	1.241 (1.158, 1.329)	0	<0.001	
Needle retention time							
≥30 min	23	2456	<0.001	1.192 (1.150, 1.236)	0	0.755	0.725
<30min	4	494	0.172	1.268 (0.902, 1.783)	90.9	<0.001	
Frequency of intervention							
≥3 times/wk	26	2895	<0.001	1.194 (1.128, 1.265)	62.3	<0.001	0.766
<3 times/wk	1	55	0.223	1.266 (0.866, 1.849)	0	–	
CG interventions							
Placebo	4	503	0.002	1.468 (1.151, 1.874)	36.3	0.194	0.264
Physical therapy	7	882	0.289	1.138 (0.981, 1.319)	82.8	<0.001	
Physical therapy & Medicine	2	294	0.001	1.261 (1.094, 1.455)	53.0	0.144	
Medicine	14	1271	<0.001	1.180 (1.128, 1.235)	0	0.825	
Follow-up:							
≥6mo	1	194	<0.001	1.354 (1.183, 1.549)	–	–	0.038^*^
<6mo	5	573	<0.001	1.346 (1.167, 1.554)	15.1	<0.001	
NR	21	2183	<0.001	1.163 (1.097, 1.234)	60.3	<0.001	

*Statistically significant.

Abbreviations: EA, electroacupuncture; TEAS, transcutaneous electrical acupoint stimulation; CW, continuous wave; 7/wkW, dilatational wave; wk, week; min, minute; mo, month.

**Fig 5:**
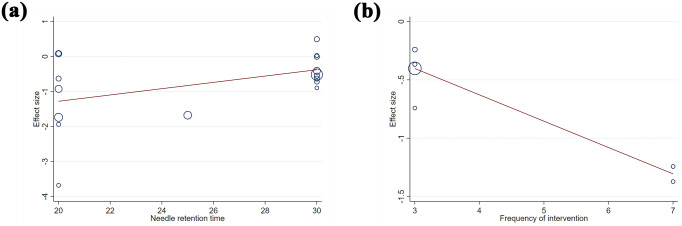
Meta-regression plots of the synthetic results. a) association between needle retention time and standardized mean difference of WOMAC Pain score; b) association between frequency of intervention and standardized mean difference of Lequesne score.

### 3.4. Secondary outcome

#### 3.4.1. WOMAC total score.

**Fig 6:**
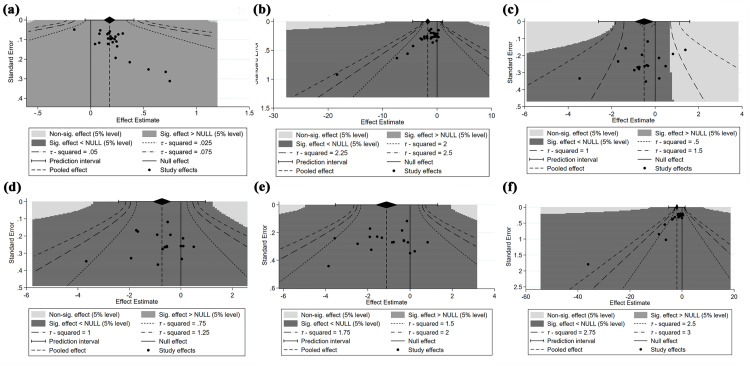
Funnel plot for the effect of EAS on KOA. a) overall response rate; b) WOMAC Total score; c) WOMAC Function score; d) WOMAC Pain score; e) WOMAC Stiffness score; f) Visual Analogue Scale.

32 RCTs [31,34,44,47,51,54,56−59,63,64,68−72,75−77,79−82,84,86−92] involving 2916 participants are included to assess the effect of EAS on WOMAC total scores. Random-effects meta-analysis demonstrates that EAS yields a large reduction in WOMAC total scores (SMD: −1.718, 95% CI: −2.192 to −1.244, P < 0.001) (Fig 3b). The evidence shows that there is significant heterogeneity between studies (I^2^ = 96.7%, P < 0.001). Sensitivity analysis (Fig 4b) reveals that excluding Lv et al. (2019) [69] changes the pooled SMD from –1.718 to –1.309, indicating moderate sensitivity to this study. Subgroup analysis (Table 4) identifies intervention duration as a heterogeneity source (P = 0.021), as studies with ≥ 4-week interventions show reduced effects (SMD = −1.248 vs. < 4-week SMD = −3.792) with reduced heterogeneity (I² = 94.7% vs. 98.7%). Meta-regression finds no significant association between prespecified covariates and heterogeneity (all P > 0.05). According to visual inspection of funnel plot, there is specific asymmetry (Fig 6b). In confirmation of our opinion, Egger’s (P < 0.001) regression tests shows that there is statistically significant publication bias.

**Table 4:** Subgroup analysis of the effect of EAS on secondary outcome.

Outcome	Subgroup title	No of trials	No of participants	P within group	SMD (95% CI)	Heterogeneity		
						I^2^ (%)	P heterogeneity	P between sub-groups
**WOMAC total score**	Overall	32	2916	<0.001	−1.718 (−2.912, −1.244)	96.7	<0.001	–
	≥4wk	25	2314	<0.001	−1.248 (−1.647, −0.848)	94.7	<0.001	0.021^*^
	<4wk	7	602	<0.001	−3.792 (−5.919, −1.665)	98.7	<0.001	
	EA	29	2728	<0.001	−1.672 (−2.167, −1.178)	96.8	<0.001	0.639
	TEAS	3	188	0.049	−2.212 (−4.409, −0.015)	97.2	<0.001	
**WOMAC function score**	Overall	18	1727	0.030	−0.509 (−0.969, −0.049)	95.0	<0.001	–
	≥4wk	15	1576	0.051	−0.526 (−1.053, 0.002)	95.8	<0.001	0.841
	<4wk	3	151	0.155	−0.443 (−1.053, 0.167)	70.5	0.034	
	EA	17	1667	0.045	−0.491 (−0.972, −0.010)	95.3	<0.001	0.376
	TEAS	1	60	0.002	−0.814 (−1.341, −0.286)	95.0	–	
**WOMAC pain score**	Overall	19	1724	<0.001	−0.735 (−1.103, −0.367)	92.0	<0.001	–
	≥4wk	15	1536	<0.001	−0.881 (−1.306, −0.456)	93.2	<0.001	0.016^*^
	<4wk	4	188	0.341	−0.183 (−0.560, 0.194)	40.2	0.170	
	EA	18	1664	<0.001	−0.741 (−1.128, −0.355)	92.5	<0.001	0.732
	TEAS	1	60	0.018	−0.628 (−1.147, −0.109)	–	–	
**WOMAC stiffness score**	Overall	18	1727	<0.001	−1.116 (−1.645, −0.587)	95.8	<0.001	–
	≥4wk	25	2706	<0.001	−1.288 (−1.888, −0.688)	96.3	<0.001	0.020^*^
	<4wk	2	244	0.403	−0.263 (−0.879, 0.353)	71.6	0.029	
	EA	17	1667	<0.001	−1.136 (−1.693, −0.579)	96.0	<0.001	0.356
	TEAS	1	60	0.004	−0.775 (−1.300, −0.250)	–	–	
**Visual Analogue Scale**	Overall	31	2533	<0.001	−1.916 (−2.443, −1.390)	96.8	<0.001	–
	≥4wk	19	1406	<0.001	−1.193 (−1.683, −0.702)	94.3	<0.001	<0.001^*^
	<4wk	12	1127	<0.001	−3.707 (−4.903, −2.511)	98.3	<0.001	
	EA	28	2355	<0.001	−2.051 (−2.630, −1.472)	97.1	<0.001	0.012^*^
	TEAS	3	178	<0.001	−1.916 (−2.443, −1.390)	68.7	0.041	
**Lequesne Score**	Overall	7	1335	<0.001	−0.695 (−0.973, −0.416)	77.5	<0.001	–
	≥4wk	5	1191	<0.001	−0.500 (−0.703, −0.298)	51.8	0.081	<0.001^*^
	<4wk	2	144	<0.001	−1.306 (−1.667, −0.945)	0	0.726	
**Lysholm Score**	Overall	9	963	0.001	1.097 (0.470, 1.725)	94.6	<0.001	–
	≥4wk	8	900	0.004	0.538 (0.175, 0.901)	84.7	<0.001	<0.001^*^
	<4wk	1	63	<0.001	12.136 (9.926, 14.345)	–	–	
	EA	8	913	0.001	1.130 (0.441, 1.819)	95.3	<0.001	0.831
	TEAS	1	50	0.001	1.031 (0.440, 1.623)	–	–	
**Peak Torque of Muscle**	Overall	5	457	0.518	0.205 (−0.417, 0.828)	90.8	<0.001	–
**Range Motion of Knee**	Overall	7	673	0.077	0.655 (−0.072, 1.382)	94.4	<0.001	–
	≥4wk	5	538	0.119	0.772 (−0.199, 1.744)	95.7	<0.001	0.418
	<4wk	2	135	0.045	0.347 (0.007, 0.687)	0.0	0.853	

*Statistically significant.

Abbreviations: EA, electroacupuncture; TEAS, transcutaneous electrical acupoint stimulation; wk, week.

#### 3.4.2. WOMAC sub-scale score.

The sub-scale scores of WOMAC are reported in 19 studies [31,34,35,40,44,47,51,54,56–58,67,68,70,71,74,80,81,91] involving 1724 participants. Among them, 18 studies [31,34,35,40,44,47,51,54,56–58,67,68,70,71,80,81,91] provide data on the WOMAC Function score, while 19 [31,34,35,40,44,47,51,54,56–58,67,68,70,71,74,80,81,91] and 18 [31,34,35,40,44,47,51,54,56–58,67,68,70,71,80,81,91] studies report the WOMAC Pain and Stiffness scores, respectively. The synthesized result (Fig 3c) indicates that: (i) Compared to the control group, EAS results in a moderate reduction in the WOMAC Function score (SMD = −0.509, 95% CI: −0.969 to −0.049, P = 0.03), a moderate-to-large reduction in the WOMAC Pain score (SMD = −0.735, 95% CI: −1.103 to −0.367, P < 0.001), and a large reduction in the WOMAC Stiffness score (SMD = −1.116, 95% CI: −1.645 to −0.587, P < 0.001); (ii) All synthesized outcomes exhibit substantial heterogeneity, as evidenced by the high I^2^ values and positive results of Cochran’s Q test for WOMAC Function (I^2^ = 95%, P < 0.001), Pain (I^2^ = 92%, P < 0.001), and Stiffness (I^2^ = 95.8%, P < 0.001), necessitating the use of a random-effects model; (iii) Sensitivity analyses (Fig 4c–e) demonstrate that the pooled effect sizes for WOMAC Function (SMD range: –0.622 to –0.342), Pain (SMD range: –0.803 to –0.781), and Stiffness (SMD range: –1.229 to –0.966) remain generally stable across leave-one-out tests; (iv) When conduct meta-regression analysis to further explain clinical and methodological heterogeneity between studies, only when needle retention time is introduced into the regression model of WOMAC Pain result as a covariable do the inter-study heterogeneity change significantly (P = 0.047) (Fig 5a); (v) Subgroup analysis (Table 4) reveals that heterogeneity exists within subgroups categorized by intervention duration (≤4 vs > 4 weeks) for pain (P = 0.016) and stiffness (P = 0.020). According to the contour-enhanced funnel plot, all results demonstrate near symmetry (Fig 6c–e). Investigation of Egger’s regression test to further assess the graph’s symmetry reveals that the results for WOMC Function (P = 0.331), Pain (P = 0.895), and Stiffness (P = 0.296) do not indicate statistically significant publication bias.

#### 3.4.3. Visual Analogue Scale (VAS).

Fig 3d demonstrates that EAS leads to a large reduction in VAS index, as evidenced by 31 studies [31,33,35,39,41–43,48,50,53,54,57,59,62–66,68–70,72,73,76,77,79,82,87–89,92] involving 2533 participants (SMD = −1.916, 95% CI: −2.443 to −1.390, P < 0.001). The combined result exhibits substantial heterogeneity (I^2^ = 96.8%, P < 0.001). Sensitivity analysis (Fig 4f) that removing Zheng et al. (2019) [69] changes the pooled SMD from –1.916 to –1.381. Meta-regression identifies no sources of heterogeneity while subgroup analysis (Table 4) reveals unresolved within-stratum heterogeneity for both intervention duration (≤4 vs > 4 weeks, P < 0.001) and intervention type (EA vs TEAS, P = 0.012). Notably, significant publication bias is observed, as demonstrated by an asymmetric funnel plot (Fig 6f) and confirmed through Egger’s regression test (P < 0.001).

#### 3.4.4. Lequesne score.

A total of 7 RCTs [31,39,45,62,78,84,92] investigating the effects of EAS on Lequesne score changes with a total of 1335 participants are analysed. The combined results (Fig 3e) of these publications indicate that EAS achieves a moderate reduction in Lequesne scores (SMD: −0.695, 95% CI: −0.973 to −0.416, P < 0.001), with evidence of heterogeneity between studies (I^2^ = 77.5%, P < 0.001). After conducting sensitivity analysis (Fig 4g), we find that excluding any single study doesn’t significantly affect the overall estimate of the influence of Lequesne score changes (SMD range: –0.773 to –0.595). Subgroup analysis (Table 4) reveals within-stratum heterogeneity when stratified by intervention duration (≤4 vs > 4 weeks, P < 0.001). The result of meta regression (Fig 5b) shows that the frequency of intervention is the source of heterogeneity (P = 0.005).

#### 3.4.5. Lysholm score.

9 studies [37,42,50,52,73,85,88,91,92] with a total sample size of 963 subjects evaluate the effect of EAS for Lysholm Score improvement is included in the meta-analysis (Fig 3f). Pooling 8 effect sizes from these studies demonstrates that EAS produces a large improvement in the Lysholm Score compared to the control group (SMD: 1.097, 95% CI: 0.470 to 1.725, P = 0.001) and there is significant heterogeneity between studies (I^2^ = 94.6%, P < 0.001). Sensitivity analysis (Fig 4h) shows that elimination of Liu et al (2012) [42] changes the pooled SMD for the Lysholm score from 1.097 to 0.538. However, in accordance with the result of meta-regression, none of the preset four covariates showed a correlation with heterogeneity. Subgroup analysis (Table 4) confirms unresolved heterogeneity exists within subgroups stratified by intervention duration (P < 0.001).

#### 3.4.6. Peak torque of muscle.

5 studies [48,57,72,85,87], encompassing 457 participants, evaluate the EAS against a control group in terms of muscle peak torque. As shown in Fig 3g, there is no significant association of EAS with peak torque of muscles (SMD: 0.205, 95% CI: −0.417 to 0.828, P = 0.518). Despite the considerable heterogeneity among the studies (I^2^ = 90.8%, P < 0.001), neither sensitivity analysis (Fig 4i, SMD range: 0.071 to 0.493) nor meta-regression identified any sources of this heterogeneity. All studies used identical EA protocols with intervention durations ≥4 weeks, precluding subgroup stratification.

#### 3.4.7. Range motion of knee.

The result of range motion of knee is available from 7 studies [32,70,83,84,87,88,92] (673 participants) and reveals no significant difference in the intervention group compared to the control group (SMD: 0.655, 95% CI: −0.072 to 1.382, P = 0.077) (Fig 3h). The studies exhibit significant heterogeneity (I^2^ = 94.4%, P < 0.001); however, attempts to pinpoint the origins of this heterogeneity through sensitivity analysis (Fig 4j, SMD range: 0.466 to 0.949), meta-regression, and subgroup analysis don’t yield any identifiable sources.

### 3.5. Safety outcomes

The adverse effects are reported in 17 studies [31,34,35,38,43,53,58,59,64,67,69,78,80,84–86,92] with a total of 2301 participants. The intervention group reports 11 types of adverse effects, while the control group reports 9 (Table 5). Skin and subcutaneous disorder is the most frequent adverse reactions in the intervention group, whereas gastrointestinal symptoms are predominant in the control group. Although most side effects are transient, tolerated by patients, or resolved with management, there is a case of withdrawal due to severe adverse reactions [35]. Besides, the summary (Fig 3i) of 9 studies involving 1,165 patients indicates a statistically higher incidence of skin and subcutaneous disorders among those receiving EAS (RR: 2.405, 95% CI: 1.564 to 3.699, P < 0.001). While in the control group, participants in 7 studies [34,38,43,53,58,59,85] experienced gastrointestinal symptoms after taking drugs, including celecoxib, aminoglucose, meloxicam, nimesulide, and diclofenac sodium.

**Table 5:** Summary of adverse effects in the intervention and control groups.

Study	Intervention group	Control group
Tu 2020 [80]	18 (Skin and subcutaneous disorders)	17 (Skin and subcutaneous disorders)
Zheng 2012 [43]	33 (Gastrointestinal discomfort and fatigued)	19 (Gastrointestinal discomfort and fatigued)
Sangdee 2002 [31]	21 (Skin and subcutaneous disorders)	NR
Gan 2018 [59]	NR	3 (Gastrointestinal discomfort)
Yin 2017 [58]	1 (Skin and subcutaneous disorders)	2 (Gastrointestinal discomfort)
Yang 2022 [85]	NR	4 (Gastrointestinal discomfort)
Zhang 2018 [64]	4 (2 skin and subcutaneous disorders, 2 vertigo)	NR
7/wkeng 2017 [53]	NR	2 (Gastrointestinal discomfort)
Wang 2022 [84]	3 (Skin and subcutaneous disorders)	NR
Fan 2011 [38]	8 (Skin and subcutaneous disorders)	14 (Gastrointestinal discomfort)
Qiu 2006 [34]	2 (Skin and subcutaneous disorders)	9 (Gastrointestinal discomfort)
Guo 2021 [78]	3 (Palpitation)	NR
Lv 2019 [69]	22 (15 skin and subcutaneous disorders, 7 acupuncture pain)	11 (7 skin and subcutaneous disorders, 4 acupuncture pain)
Zou 2018 [67]	1 (Gastrointestinal discomfort)	1 (Gastrointestinal discomfort)
Ronald 2015 [35]	4 (NR)	6 (NR)
Zhang 2022 [86]	4 (1 gastrointestinal discomfort, 1 skin and subcutaneous disorders, 2 limb discomfort)	5 (2 gastrointestinal discomfort, 1 skin and subcutaneous disorders, 2 limb discomfort)
Liu 2023 [92]	25 (5 joint swelling, 9 joint puffiness, 7 joint pain, 4 joint stiffness)	28 (8 joint swelling, 7 joint puffiness, 10 joint pain, 3 joint stiffness)

### 3.6. Trial sequential analysis

**Fig 7:**
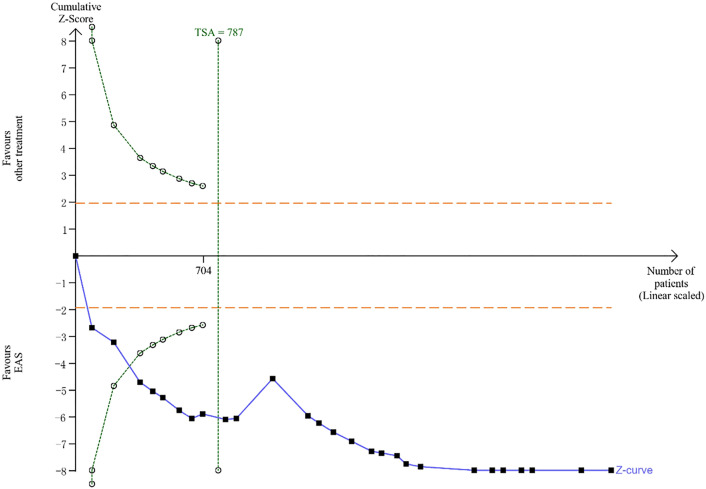
TSA for the efficacy of EAS in the treatment of KOA. A penalized test on TSA outcomes employs a significance level of α = 5% and β = 20% to control for type-I and type-II errors, respectively, with a power of 80% for adequate statistical strength, and uses a two-sided test for boundary determination.

The trial sequential analysis (TSA) of the primary outcome, derived from 27 RCTs [38,40,41,51–53,56,58,59,63,65,67,68,70,73,76–79,83,87–93] involving 2950 participants, shows that the cumulative Z-curve surpass the required information size of 787 patients (Fig 7). Furthermore, the penalized Z-curve not only crosses the conventional threshold but also surpasses the TSA threshold, confirming sufficient power. These results demonstrate robust evidence for the efficacy of EAS in KOA, surpassing potential biases from random errors and multiple testing.

### 3.7. GRADE certainty of evidence

**Fig 8:**
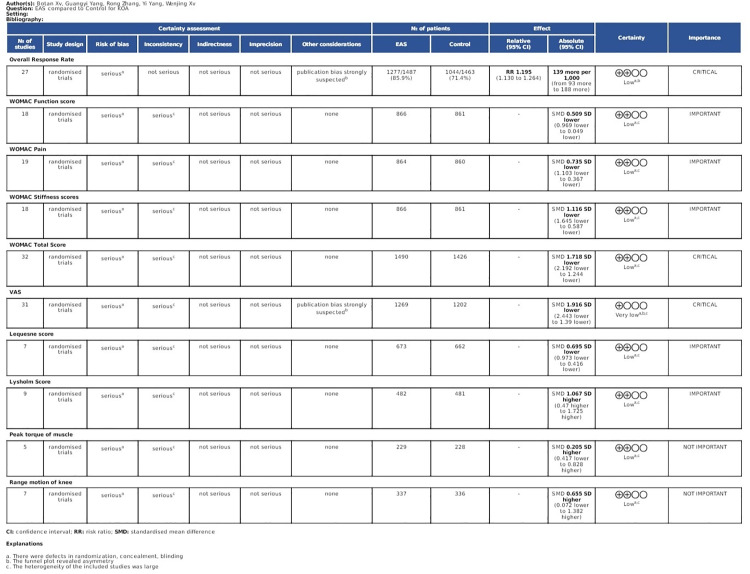
GRADE profile of EAS for KOA.

The GRADE protocol is used to access the certainty of the evidence (Fig 8). The results indicate that the overall response rate, WOMAC function, pain, stiffness, and total scores, Lequesne score, Lysholm score, peak muscle torque, and knee range of motion are of low certainty while the VAS score is of very low certainty due to extreme heterogeneity and significant publication bias. Bias risk for all outcomes is downgraded due to inadequate blinding and allocation concealment. Besides, inconsistency in most outcomes is downgraded due to significant methodological heterogeneity.

## 4. Discussion

This meta-analysis of 63 RCTs with 6475 participants indicates that EAS results in a 19.5% improvement in patient treatment efficiency (95% CI: 1.12 to 1.276; low certainty) compared to control treatments. Furthermore, the beneficial impact is reflected in the significant reduction of the WOMAC Total score by 1.718 (95% CI: −2.192 to −1.244; low certainty), aligning with decreases in the WOMAC Sub-Scale scores: 0.509 (95% CI: −0.969 to −0.049; low certainty) for function, 0.735 (95% CI: −1.103 to −0.367; low certainty) for pain, and 1.116 (95% CI: −1.645 to −0.587; low certainty) for stiffness. Similar improvements are noted in VAS, Lequesne, and Lysholm scores, with EAS decreasing the VAS score by 1.916 (95% CI: −2.443 to −1.390; very low certainty), Lequesne score by 0.695 (95% CI: −0.973 to −0.416; low certainty) and enhancing the Lysholm score by 1.097 (95% CI: 0.470 to 1.725; low certainty). However, the very high inconsistency across pooled estimates, with I² exceeding 90% for most outcomes, requires careful interpretation, and several intertwined factors seem to drive this variability. First, the trials display wide clinical diversity as the baseline disease stage covers Kellgren–Lawrence grades I to IV, and 12 studies permit concomitant medicine, all of which can influence the magnitude of response to EAS. Additionally, intervention diversity is considerable, given that only 4 trials used TEAS whereas the remainder employed EA, with stimulation parameters differing sharply in waveform, frequency (2–200 Hz), current intensity (to tolerance versus fixed mA), session frequency (2–7 per week) and treatment course (10 days-8 weeks). Our subgroup analysis shows that courses lasting at least 4 weeks and protocols using TEAS rather than EA correspond to noticeably lower heterogeneity for VAS score, suggesting that stimulation dose and modality partially moderate treatment effects. Moreover, heterogeneity is compounded by varied comparators, because 11 trials use placebo or sham, 34 employ pharmacological agents such as diclofenac or meloxicam, and 18 rely on physical therapy; subgroup analysis indicates that placebo-controlled studies yielded larger pooled SMDs, yet this difference don’t indicate a significant interaction effect between subgroups. Finally, methodological variability further inflates between-study variance, since only 2 trials are rated “good” quality while 9 are judged “weak”, and limitations such as small sample size (mean 102 participants), unclear allocation concealment in 86% of trials, and absent blinding in 90% are recognized sources of dispersion. Collectively, these observations highlight the need for standardized EAS protocols that specify acupoint selection, stimulation parameters and treatment duration, together with rigorously designed and transparently reported RCTs, to narrow heterogeneity and permit more definitive clinical inference. In contrast to the subjective improvements, no similar improvement is observed in the objective measurement, as neither the peak muscle torque nor the knee range of motion shows significant differences. While the majority of studies reported no significant difference in the incidence of adverse events, there is an increased risk of specific adverse effects in the intervention group, particularly in terms of skin and subcutaneous disorders. The risk of such adverse events is approximately 2.405 times (95% CI: 1.564 to 3.699) higher than in the control group. Clinicians should monitor patients for these potential side effects during treatment.

The efficacy and safety of EAS for KOA remains a subject of active research. Previous studies have attempted to address this issue, but the results have been inconclusive. Our findings are consistent with previous studies examining EAS as a treatment for KOA, notably those by Zeng et al. (2015), Luo et al. (2024), Chen et al. (2024), and Wang et al. (2023), who also highlighted significant improvements in pain and function. Zeng et al. (2015) conducted a network meta-analysis, revealing that electrical stimulation led to substantial reductions in pain and improved function in KOA patients [20]. Similarly, Luo et al. (2024) found significant improvements in the effectiveness of treating KOA and decreased the WOMAC pain score in patients using EA, which parallels our results, confirming the effectiveness of EAS as a non-invasive treatment [94]. Furthermore, the systematic review by Chen et al. (2024) reported improvements in KOA patients’ pain relief through high-intensity EA, similar to our study’s findings of significant reductions in pain and functional scores [95]. Additionally, Wang et al. (2023) found that EA improved the long-term WOMAC score significantly, reinforcing the notion that EAS contributes to improving the quality of life for KOA patients [96]. In contrast to our results, Ma et al. (2023) reports no significant improvement in VAS scores or treatment efficiency of EA for KOA when compares with conventional acupuncture approaches [97]. This divergence could arise from methodological variations, including differences in treatment protocols and efficacy assessment methodologies. Firstly, Ma et al. applies narrower inclusion criteria, restricting comparisons solely to EA versus manual acupuncture and excluding studies involving other control interventions such as placebo, sham acupuncture, or medication, which are included in our analysis. Secondly, their review excludes trials that combine EA with any adjunctive therapy, whereas we include such studies if both arms receive equivalent co-interventions aside from EAS. Thirdly, they conduct only pairwise meta-analyses without applying GRADE assessment or TSA, which may limit the depth and reliability of their conclusions. Lastly, they focus solely on standardized mean differences without performing subgroup or meta-regression analyses, reducing their ability to explore treatment effect moderators. In contrast to the smaller cohort in Ma et al.’s study, our larger sample size enhances statistical power. The application of the GRADE framework for evidence certainty evaluation and TSA for data adequacy assessment strengthens the validity of our conclusions. Additionally, the use of subgroup and meta-regression analyses further enables identification of potential effect modifiers, offering more nuanced clinical insights.

The therapeutic mechanisms of EAS in KOA operate through three distinct but synergistic pathways. Neural modulation occurs as EAS activates Aδ fibers, stimulating β-endorphin release, which suppresses spinal nociceptive transmission [98,99]. Simultaneously, it enhances thalamic GABAergic signaling by downregulating CB1 receptors and activating layer V pyramidal neurons, effectively alleviating central sensitization [100]. Metabolic regulation is achieved through dual control over cartilage homeostasis, wherein EAS inhibits Wnt/β-catenin-mediated MMP-13 overexpression [101,102]. and activates PI3K/Akt signaling to promote chondrocyte proliferation and delay cartilage degradation [103,104]. Immunomodulation is facilitated by both local and systemic anti-inflammatory responses, including the induction of macrophage M2 polarization, which results in an increase in CD206 + cells to suppress synovitis [105,106], and the upregulation of splenic Treg populations to establish immune homeostasis [107,108]. However, the lack of quadriceps strength improvement in our study may relate to insufficient activation of muscle satellite cells, as the EAS parameters such as current intensity and frequency, do not meet the required activation threshold [109]. Notably, most included trials applied stimulation frequencies ranging from 2 to 100 Hz and intensities of 0.5–2 mA, which are generally sufficient to activate Aδ fibers and central pain modulation mechanisms. However, in studies evaluating peak muscle torque and knee range of motion, stimulation intensity is typically adjusted based on patient tolerance rather than standardized to a defined threshold [48,70,72,85,87]. Given the considerable interindividual variability in neuromuscular excitability, this approach introduces marked heterogeneity in treatment dosing. Although patient-guided intensity adjustment enhances comfort, it could result in insufficient stimulation levels that fail to activate satellite cells or promote meaningful muscle regeneration.

Despite the established efficacy of EAS, our study observed a significantly higher incidence of skin and subcutaneous disorders in the EAS group (RR: 2.405), indicating a requirement for enhanced patient monitoring during early treatment phases. Mild skin irritations can typically be managed through proper guidance, while patient education regarding self-care techniques is crucial for mitigating these adverse effects [110]. Given the low incidence of severe adverse events and its lower gastrointestinal risk compared to NSAIDs, EAS represents a safe therapeutic option for most KOA patients, particularly those seeking non-invasive treatments to avoid systemic medication effects [111]. However, in light of the low to very low certainty of evidence as assessed by GRADE, these findings should be interpreted with caution. EAS may be considered a complementary approach, particularly for individuals with contraindications to conventional medications. Clinicians are advised to clearly communicate both the potential benefits and the increased, though generally mild, risk of skin-related adverse effects. Patient-centered decision-making remains crucial in determining the appropriateness of EAS in clinical practice.

The clinical value of this study is significant in providing an evidence-based assessment of EAS for KOA, particularly in the context of its effectiveness and safety. Through systematic analysis of 63 RCTs, this meta-analysis quantitatively demonstrates EAS’s therapeutic effects on critical endpoints, including overall response rates and standardized osteoarthritis assessment scales. In contrast to pharmacologic interventions carrying risks of chronic adverse reactions, EAS demonstrates superior safety as a non-invasive modality, showing particular effectiveness in pain management and functional recovery. The incorporation of TSA with GRADE evidence grading constitutes methodological innovation, effectively resolving previous limitations in evidence synthesis while ensuring result robustness [112]. These results support the integration of EAS into KOA treatment guidelines as a first-line non-pharmacological intervention, particularly for patients with contraindications to NSAIDs. Clinicians may consider EAS as a complementary therapy within multimodal pain management frameworks, especially for NSAID-resistant cases or patients requiring a reduced pharmacologic burden. Future research could focus on refining treatment protocols, examining long-term outcomes, and evaluating the broader applicability of EAS in different populations, including those with comorbid conditions.

## 5. Strength, differentiation, and limitation

Despite the variability in interventions and comparators, our systematic review offers a relevant and up-to-date overview of the use of EAS. The review team, composed of experts in both methodology and subject matter, adhered to contemporary guidelines for conducting and reporting systematic reviews, following a predetermined protocol with minimal deviations. We conduct an extensive search across various databases, without limiting the scope to specific languages or types of publications and assess quality of studies using valid methodological tools. Furthermore, we integrate the available data through appropriate methodologies to address issues related to improper reporting and analytical techniques observed in some trials. In addition, we conduct a range of methods including subgroup analysis, sensitivity analysis, and meta-regression analysis to examine clinical and methodological relevance and the sturdiness of our findings.

And we notice that an early pooled meta-analysis of 13 RCTs (1631 participants), published in 2021 [113], examined the efficacy of EA in the treatment of KOA and found certain advantages over analgesics for the KOA treatment. Nonetheless, the validity and clinical interpretation of the results are compromised by methodological choices related to the definition of outcomes, treatment of median values, and the exclusion of data from grey literature sources, which may result in the omission of significant evidence. Our systematic review and meta-analysis include a substantially larger number of eligible studies (n = 63) and participants (n = 6475) and evaluate a wider range of outcomes, emphasizing those definitions deemed most critical in trials assessing the efficacy for KOA. Furthermore, P.L. and colleagues analyzed only medicine-controlled trials [114]. Missing studies investigating placebo-controlled or using physical therapy which is regarded as cornerstone in managing KOA symptoms by the current treatment guideline [115] as control measure. Instead, our systematic review addresses the research question by performing distinct analyses based on the inclusion of the current clinical common control measures.

We acknowledge several limitations in both the evidence base and the review process. The majority of trials have small sample sizes, which constrains the precision of our effect estimates. Additionally, many trials are categorized as having a high or unclear risk of bias due to inadequate reporting. Specifically, most trials report numerical variables using two values before and after rather than change values, necessitating imputation for the meta-analyses. Furthermore, despite our broad search strategy, there is evidence of publication bias, with studies showing negative results often less likely to be published. Lastly, most analyses demonstrated a high degree of heterogeneity, possibly due to differences in acupoint count, follow-up periods, and treatment types. These differences should be considered when interpreting the findings.

## 6. Conclusion

Our systematic review and meta-analysis suggest that EAS may be an effective and relatively safe treatment option for KOA. However, given the low certainty of the evidence and the presence of substantial heterogeneity and potential biases, these findings should be interpreted with caution. Further high-quality, rigorously designed trials with standardized reporting are needed to validate these results and support clinical implementation.

## Supporting information

S1 Table PRISMA-2020-checklist. (DOCX)

S2 Table The search strategy for Chinese-language database. (DOCX)

S3 Table Additional study and sample characteristics. (DOCX)

S1 Dataset File Minimal data set. (ZIP)

S1 File Supplementary Materials Data Availability. (XLSX)
