# Supplementary material for: The efficacy and safety of electrical acupoint stimulation (EAS) for knee osteoarthritis (KOA): A GRADE-assessed systematic review, meta-analysis and trial sequential analysis
Source: PLoS One. 2025 Sep 25;20(9):e0331568. doi: 10.1371/journal.pone.0331568 (PMC12463219; doi:10.1371/journal.pone.0331568)
Supplement: S2 Table — (DOCX) [file pone.0331568.s002.docx]

| TableS1 The search query in Chinese database | | |
| --- | --- | --- |
| Strategy | Number | Search terms |
| Population | #1 | (膝关节 [主题词] OR膝 [主题词]) |
|  | #2 | (骨关节炎 [主题词] OR骨关节病 OR退行性关节炎 OR骨关节病, 变形) |
|  | #3 | #1 AND #2 |
|  | #4 | (骨关节炎, 膝 [主题词] OR 膝关节骨关节炎 OR 膝骨关节炎 OR 膝关节炎 OR 膝关节关节变性病) |
|  | #5 | #3 OR #4 |
| Intervention | #6 | (电刺激 [主题词] OR 电刺激疗法 [主题词] OR电刺激治疗 OR治疗性电刺激) |
|  | #7 | (针刺穴位 [主题词] OR穴位 OR 腧穴 OR 经穴 OR 针灸刺激点) |
|  | #8 | #6 AND #7 |
|  | #9 | (电针疗法 [主题词] OR 电针 [主题词] OR经皮穴位电刺激) |
|  | #10 | #8 OR #9 |
|  | **#5 AND #10** | |
