# Supplementary material for: The efficacy and safety of electrical acupoint stimulation (EAS) for knee osteoarthritis (KOA): A GRADE-assessed systematic review, meta-analysis and trial sequential analysis
Source: PLoS One. 2025 Sep 25;20(9):e0331568. doi: 10.1371/journal.pone.0331568 (PMC12463219; doi:10.1371/journal.pone.0331568)
Supplement: S3 Table — (DOCX) [file pone.0331568.s003.docx]

| **Table S3. Additional study and sample characteristics** | | | | | | | | | |
| --- | --- | --- | --- | --- | --- | --- | --- | --- | --- |
| Study | Country | Financial support | Sex | Sample size | | Trial  Duration | Means Age | | Adverse  events  reported |
|  |  |  |  | IG | CG |  | IG | CG |  |
| Tu et al 2021(80) | China | Y | M/F (M:72, F:225) | 151 | 146 | 8wk | 62.7 | 62.8 | NR |
| Zhang et al 2018(65) | China | NR | M/F (M:39, F:21) | 30 | 30 | 4wk | 40.1 | 39.5 | NR |
| Yang et al 2017(57) | China | NR | M/F (M:22, F:38) | 30 | 30 | 4wk | 55.72 | 53.49 | NR |
| Xu et al 2011(40) | China | NR | M/F (M:8, F:25) | 16 | 17 | 30d | 59.9 | 63.4 | NR |
| Zhou et al 2015(50) | China | Y | M/F (M:36, F:44) | 40 | 40 | 4wk | 54.6 | 53.8 | NR |
| Zheng et al 2012(43) | Australia | NR | M/F (M:18, F:17) | 17 | 18 | 6wk | 51.1 | 48.4 | Y |
| Wang et al 2022(83) | China | NR | M (M:68) | 34 | 34 | 4wk | 64.50 | 62.82 | NR |
| Hou et al 2020(74) | China | NR | M/F (M:6, F:30) | 18 | 18 | 3wk | 61.11 | 65.94 | NR |
| Li et al 2020(76) | China | Y | M/F (M:27, F:53) | 40 | 40 | 8wk | 55 | 53 | NR |
| Wang et al 2021(81) | China | Y | M/F (M:21, F:39) | 30 | 30 | 8wk | 64.73 | 66.10 | NR |
| Sangdee et al 2002(31) | Thailand | NR | M/F (M;22, F:73) | 48 | 47 | 4wk | 65.10 | 62.70 | Y |
| Wu et al 2015(48) | China | Y | M/F (M:36, F:64) | 48 | 47 | 4wk | 58.57 | 60.02 | NR |
| Liu et al 2018(63) | China | NR | M/F (M:43, F:57) | 50 | 50 | 3mo | 62.3 | 63.7 | NR |
| Xu et al 2019(71) | China | Y | M/F (M:97, F:103) | 100 | 100 | 8wk | 66.48 | 66.12 | NR |
| Gan et al 2018(59) | China | Y | M/F (M:31, F:29) | 30 | 30 | 4wk | 58.63 | 58.90 | Y |
| Gang et al 2016  (51) | China | Y | M/F (M:41, F:47) | 43 | 45 | 6wk | 54 | 54 | NR |
| Yin et al 2017(58) | China | Y | NR | 60 | 60 | 8wk | NR | NR | Y |
| Yin et al 2019(72) | China | Y | M/F (M:31, F:37) | 34 | 34 | 4wk | 48.23 | 49.31 | NR |
| Ji et al 2011(39) | China | NR | M/F (M:31, F:39) | 35 | 35 | 8wk | 56 | 56 | NR |
| Ju et al 2017(54) | China | Y | M/F (M:13, F:47) | 30 | 30 | 2wk | 60 | 64 | NR |
| He et al 2006(33) | China | NR | M/F (M:10, F:14) | 12 | 12 | 10d | 59.2 | | NR |
| Zhu et al 2013(44) | China | Y | M/F (M:22, F:33) | 28 | 27 | 4wk | 68.18 | 69.33 | NR |
| Zhang et al 2011(41) | China | NR | M/F (M:31, F:33) | 32 | 32 | 2wk | 42-56 | | NR |
| Jiang et al 2021(79) | China | Y | M/F (M:42, F:58) | 50 | 50 | 10d | 59.7 | 58.7 | NR |
| Yang et al 2022(85) | China | Y | M/F (M:33, F:47) | 40 | 40 | 1mo | 62.9 | 63.3 | Y |
| Su et al 2018(62) | China | NR | M/F (M:23, F:37) | 30 | 30 | 2wk | 61.27 | 60.18 | NR |
| Wang et al 2017(56) | China | Y | M/F (M:6, F:30) | 18 | 18 | 3wk | 61.11 | 65.94 | NR |
| Te Lie Ke et al 2019(70) | China | NR | M/F (M:18, F:37) | 28 | 27 | 2wk | 57.64 | 57.85 | NR |
| Liu et al 2012(42) | China | NR | M/F (M:20, F:43) | 32 | 31 | 20d | 54.0 | 55.6 | NR |
| Zhang et al 2018(65) | China | Y | M/F (M:28, F:60) | 39 | 39 | 2wk | 62.08 | 61.36 | Y |
| Huang et al 2018(61) | China | NR | M/F (M:119, F:81) | 100 | 100 | 2mo | 59.52 | 60.52 | NR |
| Deng et al 2017(53) | China | Y | NR | 40 | 40 | 2wk | NR | NR | Y |
| Zhu et al 2018(66) | China | Y | M/F (M:33, F:47) | 40 | 40 | 1mo | 56.86 | 57.07 | NR |
| Gan et al 2019(68) | China | NR | M/F (M:45, F:65) | 55 | 55 | 30d | 43-72 | 42-71 | NR |
| Zhou et al 2020(77) | China | Y | M/F (M:31, F:35) | 33 | 33 | 3wk | 65.00 | 64.80 | NR |
| Yang et al 2014(47) | China | NR | M/F (M:13, F:47) | 30 | 30 | 6wk | 66.55 | 68.10 | NR |
| Zhan et al 2019(73) | China | Y | M/F (M;23, F:27) | 25 | 25 | 4wk | 54.27 | 55.07 | NR |
| Teng et al 2022(82) | China | NR | M/F (M:29, F:33) | 31 | 31 | 4wk | 57.1 | 57.6 | NR |
| NG et al 2003(32) | China | Y | M/F (M:1, F:23) | 40 | 40 | 2wk | 84.38 | 85.88 | NR |
| Huang et al 2016(52) | China | Y | M/F (M:27, F:33) | 30 | 30 | 4wk | 40-70 | 40-70 | NR |
| Wang et al 2022(84) | China | Y | M/F (M:21, F:39) | 30 | 30 | 8wk | 62 | 62 | Y |
| Fan et al 2011(38) | China | Y | M/F (M:63, F:85) | 75 | 73 | 20d | 68.12 | 67.38 | Y |
| Ruan et al 2014(46) | China | NR | M/F (M:42, F:46) | 45 | 43 | 8wk | 54.7 | 52.4 | NR |
| Qiu et al 2006(34) | China | NR | M/F (M:9, F:51) | 30 | 30 | 4wk | 56.07 | 55.37 | Y |
| Qi et al 2017(55) | China | NR | M/F (M:48, F:77) | 65 | 60 | 60d | 55.8 | 52.5 | NR |
| Ruan et al 2014(45) | China | NR | M/F (M:39, F:33) | 38 | 34 | 3wk | 55 | 55 | NR |
| Jin et al 2020(75) | China | Y | M (M:90) | 45 | 45 | 8wk | 69.27 | 70.18 | NR |
| Liu et al 2010(36) | China | NR | M/F (M:32, F:88) | 60 | 60 | 5wk | 56 | 54 | NR |
| Guo et al 2021(78) | China | Y | M/F (M:149, F:573) | 361 | 361 | 4wk | 50.6 | 50.9 | Y |
| Lv et al 2019(69) | China | Y | M/F (M:54, F:166) | 145 | 75 | 2wk | 64.6 | 61.9 | Y |
| Zou et al 2018(67) | China | NR | M/F (M:54, F:46) | 50 | 50 | 8wk | 47.71 | 46.62 | Y |
| Yan et al 2010(37) | China | NR | M/F (M:52, F:68) | 60 | 60 | 8wk | 76.32 | 75.79 | NR |
| Ronald et al 2008(35) | UK | NR | M/F (M:13, F:55) | 34 | 34 | 5wk | 64.1 | 66.1 | Y |
| Zhang et al 2022(86) | China | NR | M/F (M:55, F:45) | 50 | 50 | 1mo | 62.37 | 63.29 | Y |
| Sun et al 2018(60) | China | NR | M/F (M:72, F:124) | 98 | 96 | 8wk | 56.3 | | NR |
| Zhou et al 2015(50) | China | Y | M/F (M:27, F:33) | 30 | 30 | 20d | 56.47 | 53.90 | NR |
| Li et al 2023(89) | China | Y | M/F (M:28, F:60) | 43 | 45 | 4wk | 57.1 | 56.8 | NR |
| Liu et al 2023(91) | China | NR | M/F (M:111, F:69) | 90 | 90 | 30d | 58.23 | 58.91 | NR |
| Duanmu et al 2023(87) | China | Y | M/F (M:36, F:44) | 40 | 40 | 4wk | 61.85 | 62.48 | NR |
| Hu et al 2023(88) | China | Y | M/F (M:26, F:34) | 30 | 30 | 6wk | 62.73 | 63.58 | NR |
| Li et al 2023(90) | China | Y | M/F (M:26, F:34) | 30 | 30 | 4wk | 71.2 | 69.1 | NR |
| Zhou et al 2024(93) | China | Y | M/F (M:69, F:93) | 81 | 81 | 4wk | 57.35 | 58.01 | NR |
| Liu et al 2023(92) | China | Y | M/F (M:138, F:132) | 135 | 135 | 2wk | 57.96 | 58.16 | Y |

Abbreviations: IG, intervention group; CG, control group; Y, yes; NR, not reported; F, Female; M, Male; d, day; wk, week; mo, month;
